# *Histone deacetylase-4* is required during early cranial neural crest development for generation of the zebrafish palatal skeleton

**DOI:** 10.1186/1471-213X-12-16

**Published:** 2012-06-07

**Authors:** April DeLaurier, Yukio Nakamura, Ingo Braasch, Vishesh Khanna, Hiroyuki Kato, Shigeyuki Wakitani, John H Postlethwait, Charles B Kimmel

**Affiliations:** 1 Institute of Neuroscience, 1254 University of Oregon, Eugene, OR, 97403, USA; 2 Department of Orthopaedic Surgery, Showa-Inan General Hospital, Akaho 3230, Komagane, 399-4117, Japan; 3 Department of Orthopaedic Surgery, Shinshu University School of Medicine, Asahi 3-1-1, Matsumoto, 390-8621, Japan; 4 Department of Health and Sports Sciences, Mukogawa Women’s University, Nishinomiya, 663-8137, Japan

**Keywords:** Cartilage, *hdac4*, Neural crest, Palate, Skeleton, Zebrafish

## Abstract

**Background:**

Histone deacetylase-4 (Hdac4) is a class II histone deacetylase that inhibits the activity of transcription factors. In humans, HDAC4 deficiency is associated with non-syndromic oral clefts and brachydactyly mental retardation syndrome (BDMR) with craniofacial abnormalities.

**Results:**

We identify *hdac4* in zebrafish and characterize its function in craniofacial morphogenesis. The gene is present as a single copy, and the deduced Hdac4 protein sequence shares all known functional domains with human HDAC4. The zebrafish *hdac4* transcript is widely present in migratory cranial neural crest (CNC) cells of the embryo, including populations migrating around the eye, which previously have been shown to contribute to the formation of the palatal skeleton of the early larva. Embryos injected with *hdac4* morpholinos (MO) have reduced or absent CNC populations that normally migrate medial to the eye. CNC-derived palatal precursor cells do not recover at the post-migratory stage, and subsequently we found that defects in the developing cartilaginous palatal skeleton correlate with reduction or absence of early CNC cells. Palatal skeletal defects prominently include a shortened, clefted, or missing ethmoid plate, and are associated with a shortening of the face of young larvae.

**Conclusions:**

Our results demonstrate that Hdac4 is a regulator of CNC-derived palatal skeletal precursors during early embryogenesis. Cleft palate resulting from HDAC4 mutations in human patients may result from defects in a homologous CNC progenitor cell population.

## Background

The vertebrate craniofacial skeleton is comprised of a complex array of cartilaginous and bony elements that must develop properly to enable efficient respiration, vocalization, and feeding. Skeletal structures of the face are derived from cranial neural crest (CNC) cells that develop at the interface of the presumptive epidermis and the neural tube, and migrate to form the pharyngeal arches, establishing progenitor populations of skeletogenic cells in the head [1,2]. Disruption of CNC cell behavior, or defects in CNC-derived tissue patterning, as might arise when function of a developmental regulatory gene is lost, can result in craniofacial disorders such as cleft palate, one of the most common birth defects in humans [3,4]. Here we examine function of a gene important for palatogenesis, *hdac4*.

Hdac4 is a class II histone deacetylase that by binds to other HDACs and myocyte enhancing factor-2 (Mef2) to inhibit transcription factor binding to target DNA [5-8]. In humans, single-nucleotide polymorphisms (SNPs) in HDAC4 are associated with non-syndromic oral clefts [9], and haploinsufficiency of HDAC4 causes brachydactyly mental retardation syndrome (BDMR) [OMIM: 600430] with associated craniofacial abnormalities [10]. The mechanism by which loss of HDAC4 causes cleft palatal disorder in humans is unknown. In mice, *Hdac4* is expressed in a number of cell types including chondrocytes, and has a critical role in regulating endochondral ossification [11]. Establishing the function of Hdac4 in craniofacial development is critical for understanding how disruption of this gene causes craniofacial skeletal defects.

Zebrafish provide a useful model for learning about craniofacial development, including understanding of the mechanisms of morphogenesis and genetic pathways that regulate the very early stages of palatogenesis [12-14]. It has been previously shown that CNC-derived palatal precursor cells migrate both rostrally and caudally to the eye to condense on the oral ectoderm, forming the palate in both mammals and zebrafish [12,13,15,16]. In both zebrafish and mammals, the palatal skeleton initiates as paired trabeculae cranii of the cartilaginous neurocranium [17,18]. Whereas mammals undergo extensive morphogenesis of the palate, including maxillary shelf formation and elevation, zebrafish eventually develop a more simplified palate, without shelves. Functionally, the anterior neurocranium of the early larva zebrafish supports the roof of the mouth, and hence is similar in function to the palate in mammals.

We recently reported that a prominent clefting of the zebrafish palatal skeleton results from loss of function mutation of *platelet-derived growth factor receptor-a* (*pdgfra*), and over-expression of the microRNA *miR-140,* which regulates the function of *pdgfra*[14]. Disruption of both genes also results in cleft palatal defects in mice or humans [19-21] demonstrating a shared genetic basis to palate formation among these species. Particularly relevant to the *hdac4* analyses we report here, clefting of the zebrafish palatal skeleton with loss of Pdgf signaling is due to a very early cellular defect. This defect involves a failure of a subset of CNC cells, those that normally generate the medial ethmoid plate, to disperse and migrate properly to reach the oral ectoderm where they condense [14].

In the present study we show that *hdac4* is required in this same Pdgfra-dependent population of CNC cells for the generation of anterior facial structures in zebrafish. Transcripts of *hdac4* are expressed in premigratory and migrating CNC cells. Morpholino mediated knockdown of *hdac4* results in an absence of anterior CNC-derived precursor cells that normally migrate medial to the eye to generate the ethmoid plate. An absence of post-migratory CNC cells along an anterior portion of oral ectoderm is subsequently associated with a shortened, clefted, or missing ethmoid plate cartilage of the palatal skeleton. Since defects in *HDAC4* in humans have been associated with mid-facial deformities, including cleft palate, understanding the function of Hdac4 in zebrafish may offer essential insights into understanding the mechanism by which this gene may normally function in the specification and/or migration of CNC cells in the development of the vertebrate face.

## Results

### Identification of *hdac4* in zebrafish and mRNA expression in the developing head

**Figure 1:**
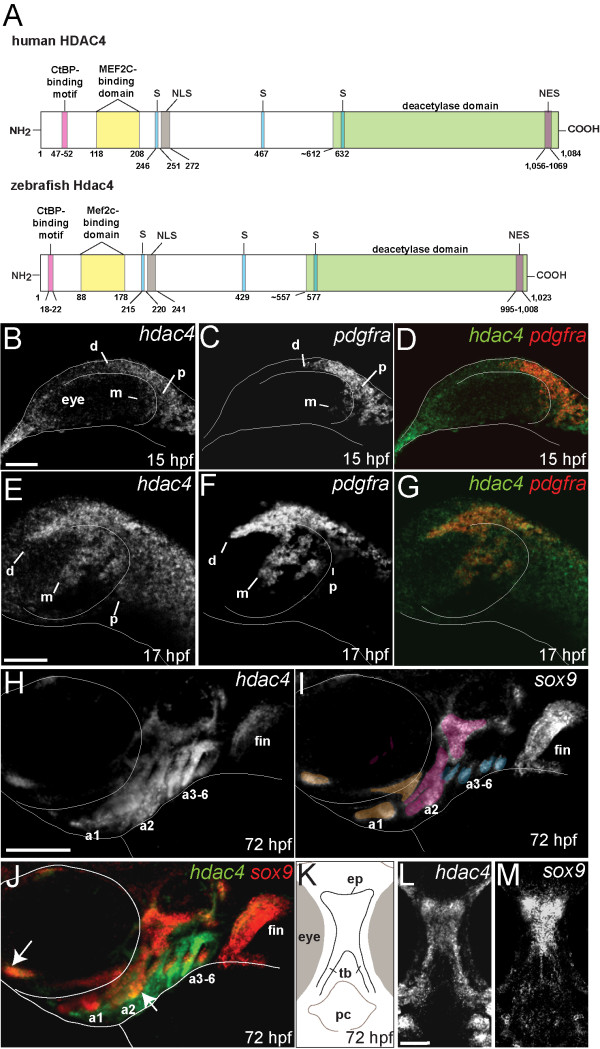
**Identification of zebrafish Hdac4 and characterization of mRNA expression in the developing head.** (**A**) Zebrafish Hdac4 protein contains functional domains conserved with human HDAC4, including nuclear localization (NLS), nuclear export (NES), and phosphorylation (S) sites [22-24,26]. (**B-J**) mRNA *in situ* hybridizations, anterior is to the left, dorsal is up. Images are projections from confocal stacks taken from the left side of the embryo. (**L** and **M**) Images are projections from confocal stacks of dissected flat-mounts, ventral side facing upwards. (**B**) *hdac4* mRNA is expressed throughout the head at 15 hpf, with expression posterior (p) and dorsal (d) to the eye. Transcript for *hdac4* is also expressed medial to the eye (m). (**C**) *pdgfra* mRNA expression at 15 hpf, posterior to the eye (p), dorsal to the eye (d), and medial to the eye (m). (**D**) *hdac4* and *pdgfra* are co-expressed at 15 hpf. (**E-G**) *hdac4* and *pdgfra* mRNAs are co-expressed at 17 hpf in three streams of expression, including dorsal to the eye (d), posterior to the eye (p), and medial to the eye (m). (**H**) At 72 hpf, *hdac4* mRNA is expressed throughout the pharyngeal arches (a1, a2, a3-6) and the pectoral fin. (**I**) *sox9* mRNA is expressed in developing cartilage (arch 1 (a1) derivatives pseudocolored in tan, arch 2 (a2) magenta, and arches 3–6 (a3-6) blue). (**J**) *hdac4* mRNA is co-expressed with *sox9* in some regions of arch 1 and 2 cartilages (indicated by white arrows), but primarily surrounds *sox9*-expressing chondrocytes in arches 3–6. (**K**) Schematic of ventral view of dissected embryo at 72hpf showing the ethmoid plate (ep) and trabeculae (tb) of the palatal skeleton, and pharyngeal cavity (pc). (**L** and **M**) *hdac4* mRNA is expressed in the ethmoid plate and trabeculae of the palatal skeleton. (**M**) *sox9* is expressed in the ethmoid plate. **B-G**: scale bar = 50 μm, H-J: scale bar = 200 μm, **L** and **M**: scale bar = 50 μm.

BLAST searches of the zebrafish genome with the human HDAC4 protein sequence returned a single closely related sequence on chromosome 9 with conservation of HDAC4 functional domains (Figure 1A, Additional file 1: Figure S1) [22-24]. Phylogenetic and syntenic analyses of the single zebrafish *hdac4* gene showed that it is orthologous to one of the duplicated *hdac4* sequences, *hdac4a,* present in several other teleosts, and orthologous to human *HDAC4* (Additional file 2: Figure S2, Additional file 3: Figure S3). Analysis with Microinspector [25] of the complete sequence of *hdac4* identifies two potential targets for the microRNA *miR-140*: one target inside exon-3, and another target spanning the end of exon-11 and beginning of exon-12 (data not shown), of interest because of previous work showing a role of this microRNA in palatal patterning [14].

RT-PCR of whole embryos revealed mRNA expression of *hdac4* as early as 4 hpf, continuing until at least 6 dpf (data not shown). Whole-mount mRNA *in situ* hybridization showed *hdac4* expression in the head at 15 hpf (hours post-fertilization) (Figure 1B). In particular, *hdac4* expression appeared strong in regions posterior and dorsal to the forming eye (Figure 1B), closely matching expression of *pdgfra,* a marker of migrating CNC cells (Figure 1C). The overlapping expression of *hdac4* and *pdgfra* (Figure 1D) suggests that *hdac4* is expressed in migrating CNC. At 17 hpf, the overlap of expression between *hdac4* and *pdgfra* was more apparent (Figure 1E-G), with expression of both genes dorsal to the eye, medial to the eye, and posterior to the eye. mRNA transcript of *hdac4* is broadly expressed throughout the head after 17 hpf, until about 72 hpf, when expression becomes localized to *sox9*-expressing cartilages in the pharyngeal arches (Figure 1H, J arrows), mesenchyme surrounding the cartilages, and the pectoral fin (Figure 1H). At this same stage, *hdac4* is also expressed in the trabeculae and ethmoid plate (Figure 1K-M).

### MO-knockdown of *hdac4* results in facial shortening and loss of palatal cartilage

**Figure 2:**
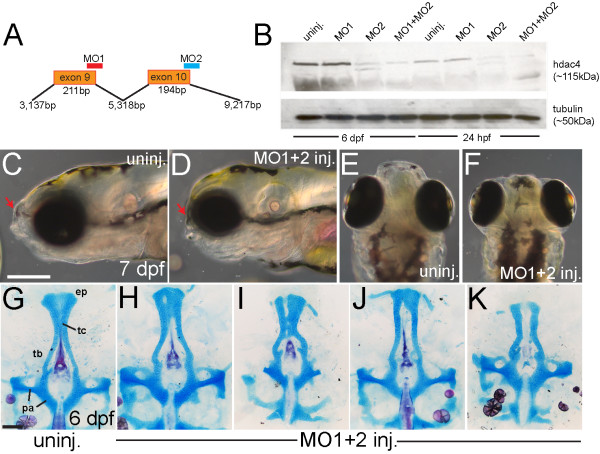
**Morpholino-knockdown of*hdac4*results in facial shortening and defects in palatal skeleton cartilage.** (**A**) Splice-blocking morpholinos MO1 and MO2 target exon-9 and exon-10 of *hdac4* mRNA. (**B**) Western blot shows down-regulation of Hdac4 protein in extracts of MO-injected embryos and larvae at 24 hpf and 6 dpf. Co-injection of MO1 (12 mg/ml) + MO2 (2 mg/ml) showed greater down-regulation of protein levels than injection of either MO alone. (**C-F**) Whole-mount images of living embryos at 7 dpf. **C** and **D** are lateral views with anterior towards the left and dorsal upwards, E and F are dorsal views with anterior upwards and dorsal is facing. (**C** and **E**) In uninjected fish, the face projects anterior to the eyes (indicated by red arrow in **C**). (**D** and **E**) The anterior projection of the face is lacking in MO-injected fish (indicated by red arrows in **C** and **D**). (**G-K**) Ventral view of Alcian Blue (cartilage) and Alizarin Red (bone) stained palatal skeletons, flat-mounted at 6 dpf. Anterior is upwards. (**G**) Uninjected fish have a normal palatal skeleton with trabeculae (tb), an ethmoid plate (ep), trabeculae communis (tc), and parachordal cartilage (pa). (H-J) MO-injected fish have a variety of palatal skeletal defects including shortened or narrowing of the ethmoid plate (H-K), holes in the ethmoid plate (**H**), clefts (**I** and **K**), and weak or absent trabeculae communii (**H-K**). **C-F**: scale bar = 250 μm, **G-K**: scale bar = 100 μm.

Co-injection of splice-blocking morpholinos, MO1 and MO2 (Figure 2A, see Methods, hereafter referred to as ‘MO-injection’), resulted in more complete protein knockdown than either MO alone (Figure 2B). At 24 hpf, almost no Hdac4 protein was detected in MO-injected embryos compared with uninjected controls (Figure 2B). By 6 dpf (days post-fertilization), protein loss was still apparent, although some expression was detected in MO-injected larvae (Figure 2B). MO-injected embryos and larvae had a distinctive facial shortening compared with uninjected controls (Figure 2C-F, red arrows in C and D). Skeletal preparations showed variable defects in palatal cartilage, predominantly including a shortening, narrowing, and loss of cartilage in the ethmoid plate or trabeculae communis (Figure 2H-K). Other palatal defects ranged from notches and holes in the ethmoid plate (Figure 2H and I), shortening of the trabeculae and ethmoid plate (Figure 2H-J), to clefting of the palatal skeleton (Figure 2J and K). In contrast, the parachordal cartilage of the posterior neurocranium was relatively unaffected (Figure 1G). Injection of a third splice-blocking MO or a translation-blocking MO generated a spectrum of palatal skeletal defects comparable with MO1 and MO2 injections (data not shown). Injection of MO targeting *hdac4* also resulted in cartilage and bone defects in the pharyngeal arches, which we are currently examining, and which may be unrelated to the palatal defects. Pharyngeal arch defects in MO-injected larvae include a gap in the hyosymplectic cartilage, and a stick-like opercle bone, which normally forms a fan-like shape (data not shown).

### Over-expression of *hdac4* mRNA rescues palatal skeleton defects associated with MO-injection and causes severe midline craniofacial defects

**Figure 3:**
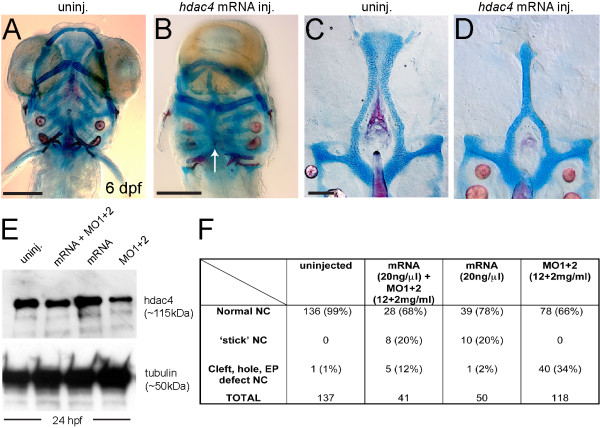
**Over-expression of*hdac4*mRNA rescues palatal defects associated with MO-injection and causes midline craniofacial defects.** (**A-D**) Alcian Blue (cartilage) and Alizarin Red (bone) stained embryos and dissected palatal skeletons. (**A** and **B**) Ventral view of whole-mount skeletal preparations of 6 dpf larvae, anterior is upwards, ventral is facing. (**C** and **D**) Flat-mounts of palatal skeletons at 6 dpf. Anterior is upwards. (**A**) Uninjected fish have normal development of craniofacial cartilage and bone. (**B**) *hdac4* mRNA injection resulted in cyclopia and a loss of normal midline patterning (indicated by white arrow). (**C**) Uninjected fish have normal palatal skeletons. (**D**) *hdac4* mRNA injection results in ‘stick’-like palatal skeletons. (**E**) Co-injection of *hdac4* mRNA (20 ng/μl) and MO1 + 2 (12 + 2 mg/ml) resulted in protein levels similar to levels in uninjected embryos 24 hours post-injection, compared with injection of mRNA or MOs alone. (**F**) Embryos used for the protein assay were also raised to 6 dpf and scored for palatal phenotypes. Co-injection of *hdac4* mRNA with MO1 + 2 resulted in a decrease of MO-like defects (i.e. cleft, hole, ethmoid plate (**EP**) defects) compared with MO-injection alone. **A** and **B**, scale bar = 200 μm; **C** and **D**, scale bar = 100 μm.

Injection of *hdac4* mRNA results in a phenotype in which midline patterning of the skeleton is impaired (Figure 3A, B). This skeletal phenotype is accompanied by cyclopia. The palatal skeleton is reduced to a single cartilage rod present in the midline (Figure 3C, D). This palatal defect, as well as cyclopia, in injected larvae matches the phenotypes of midline patterning mutants that act during or shortly after gastrulation [27,28], hence suggesting that *hdac4* over-expression is also affecting patterning at the same very early stages.

Embryos were co-injected with *hdac4* mRNA and MOs to attempt rescue of the *hdac4* MO phenotype, and test the MO specificity. Injection of *hdac4* mRNA along with *hdac4* MO resulted in Hdac4 protein levels at 24 hpf that were similar to levels detected in uninjected embryos – less than mRNA injection alone, and more than MO-injection alone (Figure 3E). Apparent differences in levels of Hdac4 protein expression in MO-injected embryos compared with uninjected embryos (Figure 3E vs. Figure 2B) are due to the inherent variability between Western blots performed on different days. Therefore, comparisons should be made within, not between these experiments.

In 6 dpf larvae, we still observed the spectrum of palatal skeletal defects characteristic of MO-only injection (as in Figure 2H-K), but these defects occurred at lower incidence as compared with MO-only injection (5/41 = 12% versus 40/118 = 34%; Figure 3F), suggesting that we obtained partial rescue of the MO phenotype. Co-injection of *hdac4* splice-blocking MO with mRNA had no effect on the incidence of the over-expression phenotype induced by the full-length mRNA (10/50 = 20% versus 8/41 = 20%). This finding was expected since the splice-blocking MO would have no effect on spliced mRNA.

### A migratory cranial neural crest cell population medial to the eye is severely reduced in *hdac4* MO-injected embryos

**Figure 4:**
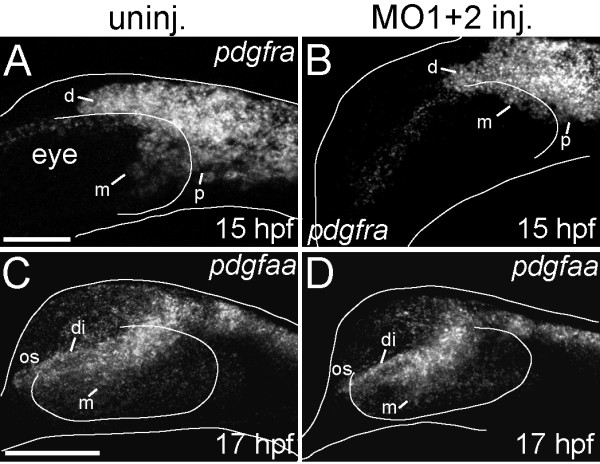
***pdgfra*mRNA expression is down-regulated in*hdac4*MO-injected embryos, although the ligand*pdgfaa*is unaffected.** (**A-D**) mRNA *in situ* hybridizations, lateral views where anterior is towards the left, dorsal is upwards, Images are projections from confocal stacks. Dorsal and ventral margins and the eye margin of the embryo were outlined from brightfield images. (**A**) Uninjected embryos showed expression of *pdgfra* dorsal to the eye (d), posterior to the eye (p), and medial to the eye (m) at 15 hpf. (**B**) MO-injected embryos showed more limited expression of *pdgfra*, in particular loss of *pdgfra* expression medial to the eye (m) at 15 hpf (compare m in **A** with **B**). (**C**) At 17 hpf, uninjected embryos showed *pdgfaa* mRNA expression in the diencephalon (di), medial to the eye (m), and in the optic stalk anterior to the eye (os). (**D**) At 17 hpf, *hdac4* MO-injected embryos had similar patterns of *pdgfaa* mRNA expression in the diencephalon (di), medial to the eye (m), and in the optic stalk (os). A and B, scale bar = 50 μm, **C-D** scale bar = 100 μm.

The palatal skeletal defects resulting from *hdac4* MO-injection are similar to the defects observed in *pdgrfra* mutants [14]. In the *pdgfra* mutant, development of the palatal skeleton is disrupted due to a defect of migration of a subset of CNC cells [14]. Resemblance of the *hdac4* palatal skeletal phenotype with that of the *pdgfra* mutant motivates the hypothesis that loss of *hdac4* also results in disruption of CNC migration. To test this hypothesis, we first examined expression of *pdgfra* itself, an excellent marker of the early CNC [14,20,29]. We observed that at the stage of migration, at 15 hpf, *pdgfra* expression appeared diminished in MO-injected embryos in a small region where cells normally are migrating medial to the eye (Figure 4A, B). This reduction was specific, for we did not detect differences in *pdgfra* expression dorsal or posterior to the eye (Figure 4A, B). In contrast, when migration is normally just beginning at 12 and 14 hpf, we observed no differences in *pdgfra* expression between MO-injected embryos and uninjected controls, (data not shown). Expression of the ligand *pdgfaa* at 15 hpf (data not shown) and 17 hpf (Figure 4C, D) appeared normal in the optic-stalk of MO-injected embryos, suggesting that the ligand for *pdgfra*-expressing CNC cells is not lost with knockdown of *hdac4*[14]. Seemingly normal *pdgfaa* expression in MO-injected embryos suggests that at least one key feature of the environment into which CNC cells migrate may not be affected by knockdown of *hdac4*, and furthermore that the defect originates within the specific population of CNC cells that migrate medial to the eye.

Sonic hedgehog (*shh*) is another factor necessary for establishing the environment for palatogenesis in zebrafish [12,13]. mRNA expression of *shh* was not altered in *hdac4*-MO injected embryos, at 10 and 14 hpf, when cells are migrating from the progenitor pool, and at 36 hpf, when cells are at the antero-ventral margin of the head (data not shown).

**Figure 5:**
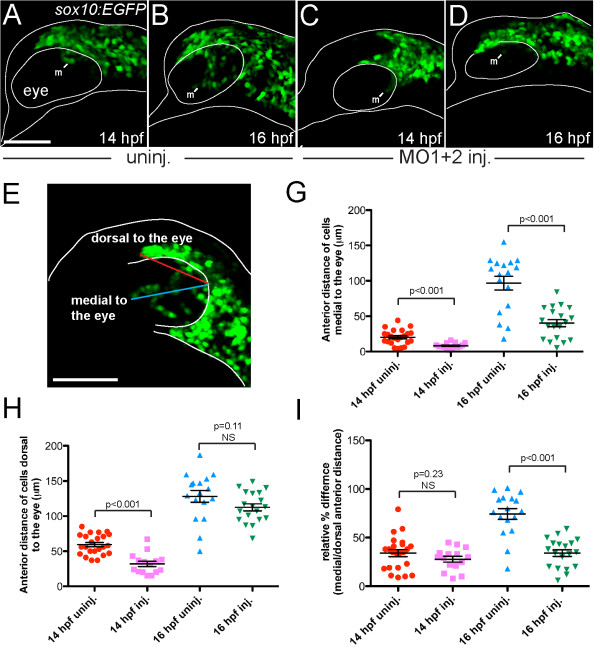
**Migratory cranial neural crest cell populations medial to the eye are reduced in MO-injected embryos.** (A-D) Live *sox10:EGFP* transgenic embryos imaged at 14 hpf (**A**, **C**) and 16 hpf (**B**, **D**). (**A-E**) Lateral views: anterior is towards the left dorsal is upwards. (**A** and **B**) Uninjected embryos (n = 6) showed migration of GFP-positive neural crest cells medial to the eye (m) starting at 14 hpf. By 16 hpf (B), cells migrated anteriorly and ventrally. (**C** and **D**) *hdac4* MO-injected embryos (n = 6) showed a reduction of GFP-positive cells medial to the eye (m). (**E**) Measurement of the maximum length of the migratory trajectories of GFP-postive cells medial and dorsal to the eye (blue line and red line, respectively). Measurements were made on fixed uninjected and MO-injected *sox10:EGFP* transgenic embryos at 14 hpf (uninjected n = 23, injected n = 15) and 16 hpf (uninjected n = 17, injected n = 19). (**G**) The absolute distance from the posterior region of the eye to the frontier of GFP-positive cells medial to the eye in MO-injected fish was significantly shorter than in uninjected controls at both 14 and 16 hpf (*p* < 0.001). (**H**) The absolute distance to the frontier of GFP-positive cells dorsal to the eye was significantly longer in uninjected controls compared to the distance in MO-injected fish at 14 hpf (*p* < 0.001), but not at 16 hpf. (**I**) The relative percentage difference of medial vs. dorsal cell migration was not significant between MO-injected embryos and uninjected controls at 14 hpf, but becomes significant at 16 hpf (*p* < 0.001), indicating that while dorsal cell migration is not affected by MO-injection, medial cell migration is specifically affected. A-E: scale bar = 100 μm. **G-I**: error bars = SEM. NS = not significant.

We used live *in vivo* imaging with the *sox10:EGFP* transgene, which is expressed by CNC [12,13], to learn whether the loss of expression of *pdgfra* was due to specific *pdgfra* down-regulation, or alternatively, to an absence of the CNC population normally migrating medial to the eye. Our findings strongly support the latter interpretation. By 16 hpf, CNC cells in control embryos migrate medial to the eye in a wedge-like pattern along the long axis of the eye, and toward the ventral and anterior margin of the eye (Figure 5B). However, in MO-injected embryos, few or no cells had migrated medial to the eye at 16 hpf (Figure 5D). To test whether the reduction or absence of cells medial to the eye in MO-injected fish was due to delayed migration, embryos were imaged up to 18 hpf, and results showed no recovery of cell populations medial to the eye even at older stages (data not shown). Matching the *pdgfra in situ* results, we observed no changes in distribution of premigratory CNC expressing the transgene in MO-injected embryos and controls at 12 hpf (data not shown), or at 14 hpf, when CNC cells first migrate medial to the eye (Figure 5A, C).

To quantify reduced or absent anterior-ward CNC cell migration and the specificity of this defect, we measured the maximum length of the migratory trajectories both medial to, and dorsal to the eye, as shown in Figure 5E. In both 14 and 16 hpf embryos, the absolute distance from the posterior region of the eye to the frontier of neural crest cells medial to the eye in MO-injected fish was significantly shorter than in uninjected controls (Figure 5G, *p* < 0.001). In 14 hpf embryos, the absolute distance to the frontier of neural crest cells dorsal to the eye was significantly longer in uninjected controls compared to the distance in MO-injected fish (Figure 5H). However, at 16 hpf, there was no significant difference in the distance of cells dorsal to the eye between MO-injected embryos and uninjected controls, suggesting that by 16 hpf, the migration of cells dorsal to the eye in injected embryos is normal (Figure 5H), whereas migration medial to the eye remained severely impaired. Such a specific defect is supported by comparing medial to the eye versus dorsal to the eye ratios: This normalized comparison of medial vs. dorsal cell migration was not significantly different when comparing MO-injected and uninjected embryos at 14 hpf but became significant by 16 hpf (Figure 5I).

To address whether cell death may have caused reduction or absence of a particular subset of migratory cells we performed Acridine Orange (AO) staining of MO-injected and uninjected embryos at 14 hpf (n = 5 MO-injected, n = 5 uninjected) and 16 hpf (n = 5 MO-injected, n = 5 uninjected). Compared with uninjected controls, MO-injected embryos did not show any localized increase in labeled degenerating cells in regions of the head populated by CNC cells fated to migrate medial to the eye and subsequently form the ethmoid plate (Additional file 4: Figure S4 and data not shown). We note that MO-injected embryos had overall higher levels of AO staining throughout the head compared to uninjected controls, likely due to non-specific effects of the morpholino.

### Reduction or absence of CNC cells in *hdac4* MO-injected embryos explains the later palatal skeletal defects

**Figure 6:**
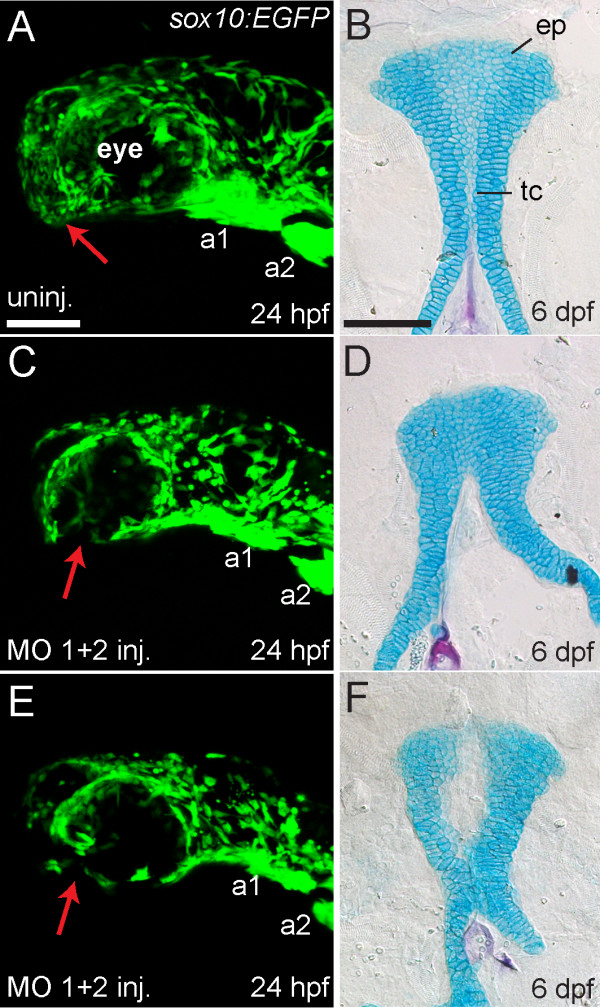
**Reduction or absence of neural crest cells in MO-injected embryos is associated with palatal defects.** (**A**,**C**, **E**) Lateral views of live *sox10:EGFP* transgenic embryos at 24 hpf. Anterior is towards the left, dorsal is upwards. (**B**,**D**,**F**) Ventral view of Alcian Blue (cartilage) and Alizarin Red (bone) stained palatal skeletons of the same individual fish shown in **A**,**C**,**E**, fixed and flat-mounted at 6 dpf. Anterior is upwards. (**A**) Uninjected embryos (n = 7) showed GFP expression in neural crest-derived tissues in the head including arch 1 (a1) and arch 2 (a2), and GFP-positive cells populate the anterior-ventral margin of the face where the palatal skeleton forms (indicated by red arrow). (**B**) Uninjected embryos had normal development of the ethmoid plate (ep) and trabeculae communis (tc) of the palatal skeletons. (**C** and **E**) *hdac4* MO-injected embryos (n = 6) showed GFP expression in neural crest-derived tissues in the head, including arch 1 (a1) and arch 2 (a2), but a lack of cells in the anterior-ventral margin of the face at 24 hpf (indicated by red arrows) (**D** and **F**) The same *hdac4* MO-injected embryos in C and E had palatal skeleton defects at 6 dpf (n = 6/6), including defects in formation of the trabeculae communis (**D**), and ethmoid plate (**F**). **A**,**C**,**E** scale bars = 100 μm, B,D,F scale bar = 100 μm.

The population of cells in *hdac-MO* injected embryos that is reduced or absent is the same population of cells fate-mapped to generate the medial ethmoid plate [12,13]. Hence, no other later-acting role of *hdac4* need be postulated to explain the ethmoid plate defects. Both the early and late phenotypes of reduced or missing CNC cells and palatal defects are variable, and if our interpretation is correct, then the severities of the early and late phenotypes should co-vary. To examine this prediction, we scored the *hdac4* MO-induced disruption of post-migratory medial ethmoid progenitors located ventral and anterior to the eye, where they are associated with oral ectoderm [13,14] in live *sox10:EGFP* and *fli1:EGFP* transgenic embryos at 24 hpf, and then scored palatal skeletal defects in these same fish at 6 dpf (Figure 6A-F). We observed, as predicted, that in all cases (n = 6/6 *sox10:EGFP* embryos, n = 9/9 *fli1:EGFP* embryos) when GFP-positive cells condensing on the oral ectoderm were not detected at 24 hpf (Figure 6C, E), cartilage defects were evident at 6 dpf (Figure 6D, F). In cases where GFP-positive cells appeared to condense normally on the oral ectoderm at 24 hpf in MO-injected embryos (n = 2/2 *sox10:EGFP* embryos), the palatal skeleton appeared normal at 6 dpf.

**Figure 7:**
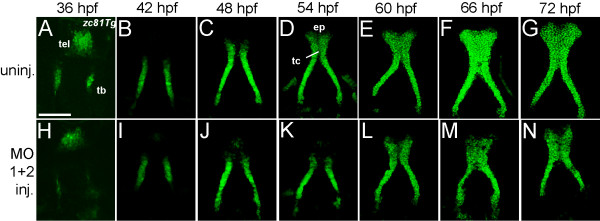
**Trabecular growth and formation of the ethmoid plate is defective in*hdac4*MO-injected embryos.** (**A**-**N**) Ventral views of *zc81Tg* transgenic embryos and larvae from uninjected (n = 3, each stage) and MO-injected (n = 3, each stage) fish. Palatal skeletons were dissected and flat-mounted. Images are projections from confocal stacks. Anterior is facing upwards. (**A**-**G**) Uninjected fish show stages of normal development of the palatal skeleton. (**A**) Trabeculae (tb) are first detected at 36 hpf as clusters of cells. At this stage, the head is bent forward and GFP expression is visible in a region anterior to the telencephalon (tel). (**B**) By 42 hpf, the trabeculae are elongated. (**C**) At 48 hpf, the anterior margin of the trabeculae converge. (**D**) At 54 hpf, the trabeculae communis forms (tc), and cells fill in the ethmoid plate (ep). (**E-G**) Between 60–72 hpf, the ethmoid plate widens (**E**) and fills in with GFP-positive cells (**F**). (**H**-**N**) MO-injected fish show defects in palatal skeleton formation. (**H** and **I**) Between 36 and 42 hpf trabeculae are smaller than those in uninjected fish at the same stage (**H**). (**J** and **K**) Trabeculae converge anteriorly, but the trabeculae communis fails to form. (**L**-**N**) Between 60–72 hpf, the trabeculae communis is formed weakly (**L**), the ethmoid plate fails to fill in with GFP-positive cells (**M** and **N**), and there is a lack of anterior growth and holes in the palatal skeleton (**M** and **N**). Scale bar = 100 μm.

Examining a cross-sectional series of stages of palatal skeletal development of *hdac4* MO-injected embryos expressing the *zc81Tg* transgene also supports our interpretation that missing CNC progenitor cells might explain the observed palatal defects (Figure 7). This transgene exquisitely and specifically labels the developing cartilages, beginning around 36 hpf and continuing for days (Figure 7A-G). At each of the stages we examined, at 6 hr intervals between 36 hpf and about 60 hpf, the trabecular cartilages of MO-injected embryos appear reduced in size, consistent with being due to a secondary effect of missing early CNC precursor cells (Figure 7 H-L). Furthermore, beginning at about 60 hpf the medial ethmoid region begins to fill in with labeled cells in uninjected controls (Figure 7E), but not completely in injected embryos (Figure 7L). Lack of cartilage at the medial ethmoid region is a feature that persists in MO-injected embryos, resulting in holes or clefts in the ethmoid plate (Figure 7M, N).

## Discussion

The palatal skeleton of the larval zebrafish is a useful model for studying genes involved in early palatogenesis in all vertebrates, including humans [14]. Here we provide evidence for a critical role of *hdac4* in a migratory or premigratory anterior population of CNC cells, the cells that eventually generate the palatal skeleton. Reduction of *hdac4* function results in embryos and larvae with shortened faces and skeletal reduction and/or clefting. Because loss of HDAC4 is associated with craniofacial defects that include oral clefts in humans [9,10], understanding the role of Hdac4 function in zebrafish palatal skeleton development is likely relevant to understanding the function of HDAC4 in human palatal development. In particular, based on our findings in zebrafish, one could well suppose that very early perturbation of CNC development could also underlie the human palatal defects.

### Defects of the palatal skeletal cartilages in *hdac4*-MO injected embryos result secondarily from early disruption of migratory or premigratory CNC cells

During normal development, the anteriorly-located CNC cells migrate in streams located posterior, medial, and dorsal to the eye, the medial cells eventually accumulating at the optic stalk at 20–24 hpf, before going on to form the medial region of the ethmoid plate, the skeletal region affected most in our *hdac4* MO-injected embryos [12-14]. We find that at 16 hpf, *hdac4* MO-injected embryos showed either a complete absence of, or significantly fewer, CNC cells migrating medial to the eye compared to uninjected controls. There is no evidence that the cells are present, and then disappear, and it remains unclear if cells are lacking in the pre-migratory CNC pool posterior to the eye, which includes other skeletal precursor cells (e.g., the trabecular precursors), or if cells simply fail to migrate. All subsequent cartilaginous defects likely result from the reduction or absence of this early population of progenitor cells: In 24 hpf *hdac4* MO-injected fish, as expected from the early defect, post-migratory CNC cell populations were greatly reduced in the region of the anterior oral ectoderm, and by 6 dpf these same fish displayed defects in the palatal skeleton itself.

In mouse, loss of *Hdac4* causes no apparent loss of chondrocytes, but premature chondrocyte hypertrophy, resulting in early onset ossification of cartilage [11]. In zebrafish, we observe an early absence of specific CNC cells corresponding to a later loss of chondrocytes in the ethmoid plate region of the palate, strongly arguing that the origin of the defect in zebrafish is due to missing cells and not a defect in cartilage matrix production. Reduction or absence of post-migratory CNC cells in the anterior-most region of the forming palatal skeleton of *hdac4* MO-injected embryos at 24 hpf is consistent with our proposal that the developmental defect occurs during the migratory or premigratory stages. We also observed *hdac4* transcripts in the chondrocytes of the ethmoid plate (as well as other cartilages) at 72 hpf. The earlier requirement in the CNC itself, however, is sufficient to fully explain the palatal defects we observed. We also note that whereas the palatal defects are the most prominent ones observed, it is unlikely that this is the only deficiency. For example, MO-injected fish appear to have shorter faces overall, suggesting additional roles for Hdac4 in facial patterning.

Whereas we find that loss of *hdac4* results in a CNC defect as early as 16 hpf, there are several possibilities as to what the exact defect(s) may be. As with loss of function of Pdgfra [14], the defect seems specific, or nearly so, for cells which normally migrate in a pathway medial to the eye. Hence one interpretation of our finding is these CNC cells lack the ability to recognize chemotactic or other cues that mark this pathway selectively. Alternatively, the defect could be further upstream, and result in the cell subset being improperly specified, or to be missing due to cell death. AO staining suggests that death of this specific migratory CNC cell population, or perhaps death of pre-migratory cells, is an unlikely mechanism causing the absence of cells.

Further work will be required to resolve this issue; at present critical markers labeling specific subsets of CNC cells are not available. The presence of some cells medial to the eye in our *hdac4* MO-injected embryos suggests that CNC cells are capable of migrating correctly. However, their presence might well be due to the fact that our MOs do not knock down *hdac4* completely. If so, then a model with complete loss of *hdac4* function should result in absence of all CNC cells medial to the eye, and more severe palatal skeletal defects than we observed.

### Gene targets of Hdac4 involved in craniofacial development

The gene targets of Hdac4 required in the CNC are unknown. Although established for CNC cell migration in the formation of the palatal skeleton [14], based on the results of this analysis, *pdgfra* and *pdgfaa* are not direct transcriptional targets of Hdac4 repression. Future investigation, by transcriptional screening or protein co-immunoprecipitation may identify targets Hdac4 required for zebrafish palatal skeletal development. In particular, targets of CNC cell specification or migration would help elucidate the cause of the early CNC cell defect.

An established target of repression by Hdac4 is Mef2c [7,8]. Loss of *Mef2c* in mice does not appear to result in defects involving the palatal skeleton or palate [30,31], although mice with loss of a single copy of both *Mef2c* and the *Distal-less* patterning genes *Dlx5/6* suffer cleft palate [31]. Although zebrafish *mef2ca* mutants have no palatal defects (unpublished results), research is currently underway to understand the relationship between Hdac4 and *mef2c genes* (duplicated in zebrafish)*,* in combination with *dlx* genes in craniofacial development. Loss of *mef2ca* and *dlx* genes cause other defects to the craniofacial skeleton in zebrafish [32,33] although analysis of their combined loss on palatal patterning is unknown.

In zebrafish, over-expression of the inhibitory micro-RNA *miR-140* results in palatal skeleton reduction [14]. *miR-140* has also been identified as a repressor of *Hdac4* in mouse [34], although Eberhart et al. (2008) did not identify any *miR-140* binding sites in the 3′-UTR of zebrafish *hdac4*. Recent studies have challenged conventional wisdom that microRNAs only bind to the 3′UTR of genes by showing that microRNAs can also function by binding to targets in the coding sequence of genes [35]. Although two potential targets for miR-140 were identified for *hdac4*, clearly, any mechanistic function of *miR-140* inhibiting *hdac4* requires experimental testing. If *miR-140* has an additional function to repress *hdac4* independent of *pdgfra* repression, it is possible that splice-inhibiting MO-induced down-regulation of *hdac4* mRNA by MO could result in less target for *miR-140*, thus leading to the availability of excess of *miR-140* that could repress other target genes, leading to palatal skeleton defects.

Development of the palatal skeleton in zebrafish and the mammalian palate involves control of CNC cell migration and condensation by many of the same gene pathways, including the *pdgf* and *shh* pathways [12-14]. We have demonstrated that Hdac4 is another important gene involved in the regulation of a subset of CNC cells that form the palatal skeleton in zebrafish. Further understanding of the mechanism of Hdac4 function, and analysis of targets of Hdac4 activity will generate a more complete model of how Hdac4 controls development of the palatal skeleton, and should further inform understanding of how loss of HDAC4 in humans causes craniofacial disorders.

## Conclusions

Knockdown of Hdac4 by morpholino results in the reduction or absence of a specific population of migratory CNC cells in the zebrafish head that normally contribute to the formation of the anterior palatal skeleton. Reduction or absence of migratory cells, detected by 16 hpf, corresponds with a reduction in CNC-derived antero-ventral cells of the face by 24 hpf, and then reductions of ethmoid plate cartilage, evident as early as 54 hpf. By 6dpf, the ethmoid plate is shortened, clefted or missing. The results of this study offer insights into the mechanism of how Hdac4 normally functions in regulating early CNC cell behavior in craniofacial skeletogenesis. Since defects in *HDAC4* in humans are associated with cleft palate, understanding the function of this gene in the normal specification and migration of CNC cells may reveal how loss of *HDAC4* causes craniofacial malformations.

## Methods

### Fish maintenance and transgenic zebrafish

Fish were raised, staged, and euthanized according to established protocols [36,37]. All procedures were approved by The Institutional Animal Care and Use Committee (IACUC) at the University of Oregon. AB-strain fish were used for wild-type analysis and for morpholino (MO) and mRNA injections. MO-injected embryos were stage-matched with wild-type embryos by somite number or other developmental criteria [37]. The transgenic lines Tg*(−4.9sox10:EGFP)*^*ba2*^ and Tg(*fli1:EGFP)*^*y1*^ label neural crest cells [13,38]. *zc81Tg* is a cartilage-specific transgenic discovered while making the Tg(*foxp2-enhancerA:EGFP)*^*zc42*^ transgenic [39].

### Identification and sequence analysis of hdac4 in zebrafish

Zebrafish *hdac4* cDNA sequence was previously published in NCBI [Genbank: NW_001879481]. We originally identified a partial genomic sequence of *hdac4* by alignment of the zebrafish genomic sequence with full-length *hdac4* sequences of other vertebrate species using ensembl zv7 [Ensembl: ENDARG00000041204]. Using Ensembl BLAST, we identified 3,518 bp of zebrafish *hdac4*, but not the full-length gene sequence as reported for other species. To identify the 5′ end of the gene, we used 5′ RACE (Rapid Amplification of cDNA Ends) to identify a further 486 bp at the 5′ end of the gene including the start codon (GeneRacer, Invitrogen). Full-length *hdac4* located on chromosome 9 is identified in Ensembl zv8 and zv9 versions of the zebrafish genome sequence. Alignment of Hdac4 protein sequence in zebrafish with human HDAC4 was performed using SIM alignment software [40]. MicroInspector [25] software was used to identify potential targets for the microRNA *miR-140 in hdac4*.

### Morpholino and mRNA design and injections

Gene Tools supplied morpholino oligonucleotides targeting *hdac4*. Two splice-inhibiting morpholino oligonucleotides were designed that targeted the junction of exon-9 to intron-9 (MO1: ATTTGTTATGCCAGCGACTGACCTG) and exon-10 to intron-10 (MO2: AGAGCCACAGAGGAGCTGCTTTACC) (see Figure 2A). One or two-cell stage embryos were injected using approximately 3 nl of morpholino solution. Co-injection of both MOs (MO1 = 12 mg/ml + MO2 = 2 mg/ml) was much more effective at knocking down mRNA splicing than either MO singly (see Figure 2B). Morpholinos were tested at a range of concentrations. The final concentration of the combined MO1 + MO2 dose gave the maximum penetrance of phenotypes with minimal lethality. A third splice-inhibiting MO targeting the junction of exon-2 with intron-2 (MO3: AATCCCAGCAGCCTCACCTTGACAT), and a translation-inhibiting MO targeting exon-1 (MOTB: AGCGCCACACTCACATCAACCATCA) were also tested. A standard control MO provided by Gene Tools (CCTCTTACCTCAGTTACAATTTATA) was injected in parallel with injection of *hdac4*-targetting MOs, and *hdac4* MO-like phenotypes were not observed in control-injected larvae.

Full-length cDNA of zebrafish *hdac4* cloned into pBluescript KS + (Invitrogen) provided template for making sense mRNA for over-expression and rescue experiments. mRNA was synthesized using the mMessenger mMachine SP6 kit (Ambion) according to the manufacturer’s protocol. For over-expression of *hdac4* mRNA, 40 ng/ul of full-length *hdac4* mRNA in a 3 nl volume was injected into one-cell-stage embryos. For the rescue assay, *hdac4* MO1 + 2 (12 + 2 mg/ml) and *hdac4* mRNA (20 ng/ul) was injected into one-cell stage embryos in a combined 3 nl volume. For the rescue assay, doses of mRNA higher than 20 ng/ul resulted in early lethality when co-injected with the MOs.

### RT-PCR and immunodetection by Western blot

Total RNA was extracted from whole embryos and larvae between 4 hpf and 6dpf. 30 embryos/larvae at each stage were dissolved in 1 ml Trizol (Invitrogen) and frozen at −80°C. mRNA was extracted according to the manufacturer’s instructions and resuspended in 20 μl of pure H_2_O with 0.5 μl RNAse inhibitor (Roche). RT-PCR was performed using the Superscript III kit (Invitrogen) using OligoDTs according to the manufacturer’s protocol. PCR was performed using standard protocols and reagents. The efficiency of splice-inhibition from MO1 was tested using primers designed to exon-8, exon-10 and intron-9. For MO2, primers were designed to exon-9, exon-11, and intron-10 (see Additional file 5: Figure S5).

For immunodetection by Western blotting, injected embryos and uninjected controls were harvested at 24 hpf and 6 dpf. At 24 hpf, 30 anesthetized embryos were de-chorionated and pooled together in 100 μL SDS loading buffer (0.63% 1 M Tris–HCl, pH 6.8, 1% glycerol, 0.5% beta-mercaptoethanol, 35% SDS). At 6dpf, 30 anesthetized embryos were pooled in 200 μL SDS buffer. Embryos were boiled for 5 min. and stored at –80°C until use. Twenty micrograms of each protein was separated by SDS-PAGE and immunodetected using a polyclonal antibody raised against amino acids 530–631 of human HDAC4 (H-92, sc-11418, Santa Cruz Biotechnology). Proteins from SDS-PAGE gels were transferred to Immobilon P membrane (Millipore), and membranes were blocked by incubation with 4% non-fat dry milk in Tris-buffered saline (TBS) at 4°C overnight. The blot was then incubated in 4% non-fat dry milk in TBS, 0.05% Tween 20 (TBST) containing HDAC4 antibody. Goat anti-rabbit HRP-conjugated antibody was used as a secondary antibody (Pierce). ECL plus Western blotting Detection System (Amersham Biosciences) and X-OMAT AR film (Eastman Kodak Company) were used to detect signal.

### Tissue labeling and mounting

Fixed 6dpf larvae were double stained using Alcian Blue (cartilage) and Alizarin Red (bone) using published protocols [41]. Palatal skeletons stained with Alcian Blue and Alizarin Red and palatal skeletons from transgenic animals were dissected and flat mounted as described [42]. We performed whole-mount fluorescent mRNA *in situ* hybridization as described [33], using previously described probes: *hdac4*[43], *pdgfra*[14], *pdgfaa*[14]. Whole-mount embryos and larvae were mounted intact using 3% methylcellulose and 1.2% agarose on standard glass slides and coverslips. To identify cell death, embryos were incubated for 20 min. in 5 μg/ml Acridine Orange (AO, Sigma) dissolved in embryo medium in the dark. Embryos were then washed three times in embryo medium and mounted in 0.5% agarose in tissue culture dishes with cover glass bottoms (World Precision Instruments).

### Whole-mount and *in vivo* imaging

For imaging both Alcian Blue and Alizarin Red stained samples and fluorescent transgenic palatal skeletons, palatal skeletons were dissected from the rest of the craniofacial skeleton and flat-mounted. For *in vivo* CNC cell migration studies *sox10:EGFP* transgenic embryos were mounted laterally on coverslips using 3% methylcelluose and 1.2% agarose and sealed in chambers using vacuum grease. Embryos were staged according to somite number, and scanned by confocal microscopy approximately every 2 hours. Between scans, embryos were stored at 28.5°C in their sealed chambers.

### Image analysis and statistics

Measurements were made on images using Zeiss AIM software. Statistics were performed using Excel (Microsoft) and Prism (GraphPad) software.

### Phylogenetic and conserved synteny analyses

Phylogenetic and conserved synteny analysis was used to validate *hdac4* orthology and to verify that it is present in single copy in the zebrafish genome [44-48]. A phylogenetic tree of vertebrate Hdac4 and outgroup (human HDAC5 and HDAC9) proteins was inferred using the Phylogeny.fr platform [49] including sequence alignment by MUSCLE [50], Maximum Likelihood phylogeny generation with PhyML (JTT + G + I model of protein evolution, 100 bootstrap replicates[51]) and tree drawing with TreeDyn [52]. Accession numbers are given in Additional file 1: Figure S1. Conserved synteny analyses were performed using the Synteny Database [53].

## Competing interests

The authors declare that they have no competing interests.

## Authors’ contributions

AD carried out morpholino injections, analysis of transgenics, skeletal preparations, molecular genetics studies, protein sequence analysis, and wrote the first draft of the manuscript. YN performed Western blot experiments. IB and JHP carried out phylogenetic and syntenic analyses. VK performed *in situ* hybridizations for *pdgfra*. HK and SW assisted YN with protein assays. CBK participated in the design, coordination and interpretation of experiments and helped to draft the manuscript. All authors read and approved the final manuscript.

## Supplementary Material

Additional file 1 **Figure S1.**Protein alignment of human HDAC4 with zebrafish Hdac4. Functional domains are shaded as follows: CtBP-binding domain (pink), MITR-binding domain (yellow), serine residues associated with nuclear export by by chaperone 14-3-3 (blue), nuclear localization signal (grey), deacetylase domain (green), nuclear export signal (purple). Alignment was performed using SIM Alignment software. Click here for file

Additional file 2 **Figure S2.**Maximum likelihood phylogeny of vertebrate Hdac4 proteins. Phylogenetic analysis of the single *hdac4* gene in zebrafish showed that it is orthologous to one of the duplicated *hdac4* sequences found in the genomes of several other teleosts, including medaka, stickleback and pufferfish. The tree is rooted with human HDAC5 and HDAC9 proteins encoded by genes paralogous to *HDAC4* and along with *HDAC7*, arising in the vertebrate R1 and R2 rounds of genome duplication. GenBank/ENSEMBL accession numbers are given for each sequence. Bootstrap values of 100 pseudoreplicates are shown; nodes with support below 50% have been collapsed. The position of the single zebrafish Hdac4 protein is ambiguous, but was assigned as an ortholog of the teleost Hdac4a proteins based on conserved synteny data (Additional file 3: Figure S3). Although the *hdac4b* gene is present in the medaka genome (scaffold279), its partial sequence was too short to be included in the phylogenetic reconstruction. Click here for file

Additional file 3 **Figure S3.**Conserved synteny analyses of teleost *hdac4* genes. Zebrafish *hdac4* is adjacent to *twist2* on linkage group 9, which reflects the location of human *HDAC4* adjacent to *TWIST2*. The next most closely conserved sequence between zebrafish and human was an unannotated *hdac7a*-related pseudogene on linkage group 23 in zebrafish, adjacent to *twist3*. A) Dotplot of the zebrafish (Dre) *hdac4* gene region on chromosome Dre9 (X axis) compared to the stickleback (Gac) genome (Y axis). The zebrafish *hdac4* region shares extensive conserved synteny with the *hdac4a* region on stickleback chromosome groupXVI, but substantially less with the *hdac4b* region on groupI. Thus, the single *hdac4* gene in zebrafish is *hdac4a*. B) Dotplot of the human (Hsa) *HDAC4* gene on chromosome Hsa2 compared to the stickleback genome. The human HDAC4 regions shares extensive conserved synteny with both *hdac4* regions in stickleback (groupXVI and groupI). The pufferfish and medaka genomes show a similar relationship (not shown), providing strong evidence for the generation of teleost *hdac4* duplicates during the course of the teleost-specific genome duplication. (C) Dotplot of the human *HDAC4* gene on Hsa2 compared to the zebrafish genome. Conserved synteny is shared with the *hdac4a* region on Dre9 as well as with Dre6 and Dre2. A second *hdac4* gene, however, is not found on Dre6 nor on any other zebrafish chromosome suggesting that *hdac4b* has been lost in the zebrafish lineage. (D) Dotplot of the stickleback genomic region on linkage groupI (GacgroupI) surrounding the *hdac4b* vs. the stickleback genomes (Dre chromosomes) showing that *hdac4b* in zebrafish was likely on Dre1 or Drfe6 before it was lost. Click here for file

Additional file 4 **Figure S4.**bCell death is not increased in MO-injected embryos in regions where medially-migrating CNC cells are present. (A and B) Live embryos stained with AO and imaged at 14 hpf. Lateral views: anterior is towards the left dorsal is upwards. Images are projections from confocal stacks. MO-injected embryos had overall higher levels of AO staining throughout the head compared to uninjected controls. MO-injected embryos did not show any localized increase in labeled degenerating cells in regions of the head populated by CNC cells fated to migrate medial to the eye and subsequently form the ethmoid plate. Scale bar = 100 μm. Click here for file

Additional file 5 **Figure S5.***hdac4* mRNA splicing is down-regulated in MO-injected embryos. RT-PCR of cDNA from whole embryo RNA extractions at 24 hpf and 3 dpf. Combined injection of MO1 and MO2 resulted in down-regulation of normal splicing between exon-8 and exon-9 and exon-9 and exon-10 (e8e10 = PCR product showing splicing between exon-8 through exon-10 splicing; e9e11 = PCR product showing splicing between exon-9 through exon-11; e8i9 = PCR product showing mis-splicing resulting in inclusion of intron between exon-8 and intron-9; e9i10 = PCR product showing mis-splicing resulting in inclusion of intron between exon-9 and intron-10). The expression of mRNA with intronic sequence was higher in injected embryos than in uninjected controls. RT-PCR for β-*actin* (control) was performed to demonstrate total mRNA levels used for RT-PCR were equal between samples. Click here for file
