# Supplementary figures and images for: Histone deacetylase-4 is required during early cranial neural crest development for generation of the zebrafish palatal skeleton
Source: BMC Dev Biol. 2012 Jun 7;12:16. doi: 10.1186/1471-213X-12-16 (PMC3426487; doi:10.1186/1471-213X-12-16)

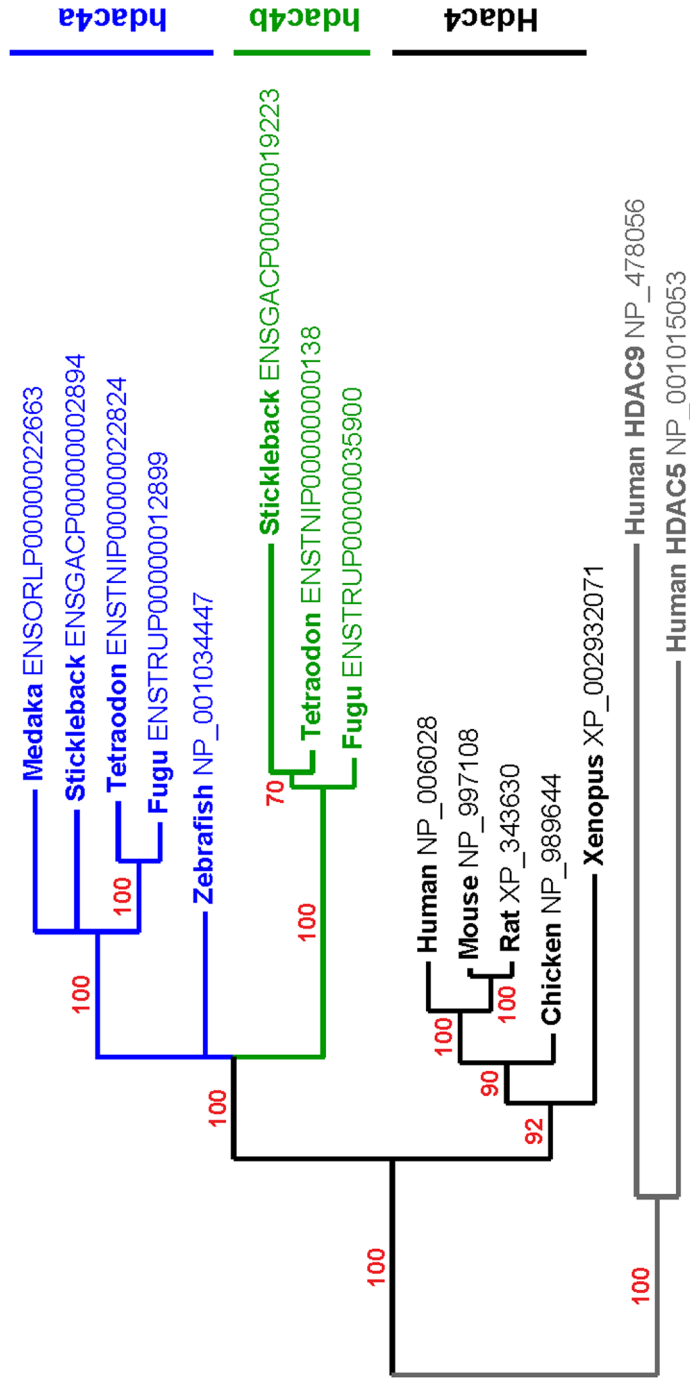

Supplement: Additional file 2 — Figure S2.Maximum likelihood phylogeny of vertebrate Hdac4 proteins. Phylogenetic analysis of the single hdac4 gene in zebrafish showed that it is orthologous to one of the duplicated hdac4 sequences found in the genomes of several other teleosts, including medaka, stickleback and pufferfish. The tree is rooted with human HDAC5 and HDAC9 proteins encoded by genes paralogous to HDAC4 and along with HDAC7, arising in the vertebrate R1 and R2 rounds of genome duplication. GenBank/ENSEMBL accession numbers are given for each sequence. Bootstrap values of 100 pseudoreplicates are shown; nodes with support below 50% have been collapsed. The position of the single zebrafish Hdac4 protein is ambiguous, but was assigned as an ortholog of the teleost Hdac4a proteins based on conserved synteny data (Additional file 3: Figure S3). Although the hdac4b gene is present in the medaka genome (scaffold279), its partial sequence was too short to be included in the phylogenetic reconstruction. [file 1471-213X-12-16-S2.pdf]

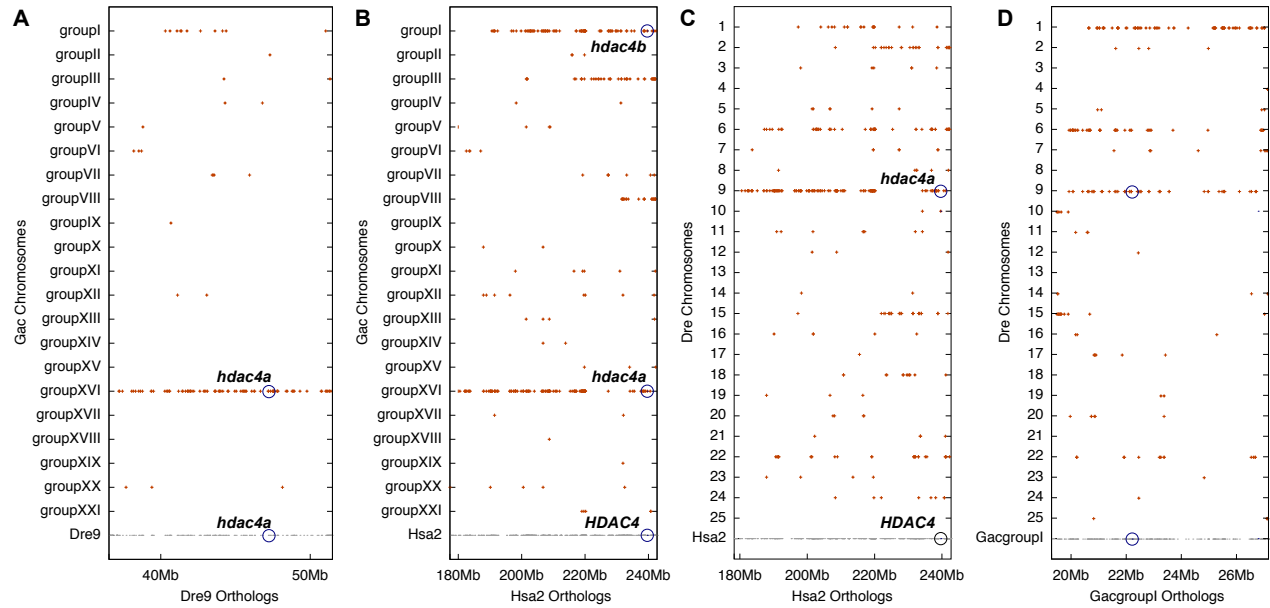

Supplement: Additional file 3 — Figure S3.Conserved synteny analyses of teleost hdac4 genes. Zebrafish hdac4 is adjacent to twist2 on linkage group 9, which reflects the location of human HDAC4 adjacent to TWIST2. The next most closely conserved sequence between zebrafish and human was an unannotated hdac7a-related pseudogene on linkage group 23 in zebrafish, adjacent to twist3. A) Dotplot of the zebrafish (Dre) hdac4 gene region on chromosome Dre9 (X axis) compared to the stickleback (Gac) genome (Y axis). The zebrafish hdac4 region shares extensive conserved synteny with the hdac4a region on stickleback chromosome groupXVI, but substantially less with the hdac4b region on groupI. Thus, the single hdac4 gene in zebrafish is hdac4a. B) Dotplot of the human (Hsa) HDAC4 gene on chromosome Hsa2 compared to the stickleback genome. The human HDAC4 regions shares extensive conserved synteny with both hdac4 regions in stickleback (groupXVI and groupI). The pufferfish and medaka genomes show a similar relationship (not shown), providing strong evidence for the generation of teleost hdac4 duplicates during the course of the teleost-specific genome duplication. (C) Dotplot of the human HDAC4 gene on Hsa2 compared to the zebrafish genome. Conserved synteny is shared with the hdac4a region on Dre9 as well as with Dre6 and Dre2. A second hdac4 gene, however, is not found on Dre6 nor on any other zebrafish chromosome suggesting that hdac4b has been lost in the zebrafish lineage. (D) Dotplot of the stickleback genomic region on linkage groupI (GacgroupI) surrounding the hdac4b vs. the stickleback genomes (Dre chromosomes) showing that hdac4b in zebrafish was likely on Dre1 or Drfe6 before it was lost. [file 1471-213X-12-16-S3.pdf]

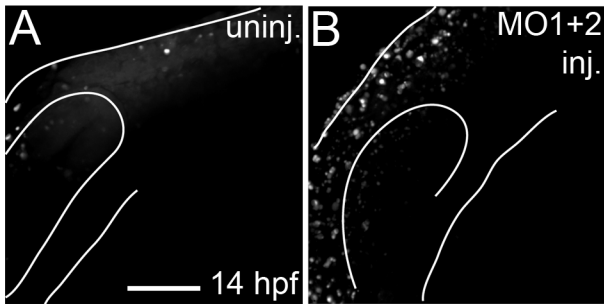

Supplement: Additional file 4 — Figure S4.bCell death is not increased in MO-injected embryos in regions where medially-migrating CNC cells are present. (A and B) Live embryos stained with AO and imaged at 14 hpf. Lateral views: anterior is towards the left dorsal is upwards. Images are projections from confocal stacks. MO-injected embryos had overall higher levels of AO staining throughout the head compared to uninjected controls. MO-injected embryos did not show any localized increase in labeled degenerating cells in regions of the head populated by CNC cells fated to migrate medial to the eye and subsequently form the ethmoid plate. Scale bar = 100 μm. [file 1471-213X-12-16-S4.pdf]

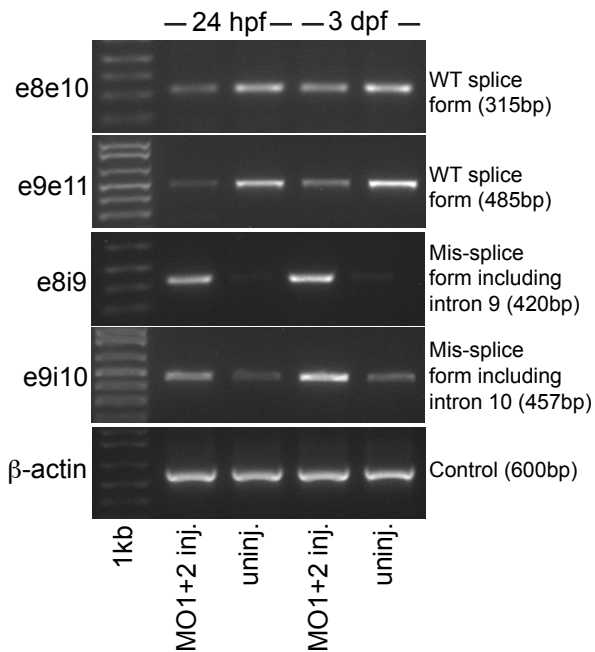

Supplement: Additional file 5 — Figure S5.hdac4 mRNA splicing is down-regulated in MO-injected embryos. RT-PCR of cDNA from whole embryo RNA extractions at 24 hpf and 3 dpf. Combined injection of MO1 and MO2 resulted in down-regulation of normal splicing between exon-8 and exon-9 and exon-9 and exon-10 (e8e10 = PCR product showing splicing between exon-8 through exon-10 splicing; e9e11 = PCR product showing splicing between exon-9 through exon-11; e8i9 = PCR product showing mis-splicing resulting in inclusion of intron between exon-8 and intron-9; e9i10 = PCR product showing mis-splicing resulting in inclusion of intron between exon-9 and intron-10). The expression of mRNA with intronic sequence was higher in injected embryos than in uninjected controls. RT-PCR for β-actin (control) was performed to demonstrate total mRNA levels used for RT-PCR were equal between samples. [file 1471-213X-12-16-S5.pdf]
