# Supplementary material for: Histone deacetylase-4 is required during early cranial neural crest development for generation of the zebrafish palatal skeleton
Source: BMC Dev Biol. 2012 Jun 7;12:16. doi: 10.1186/1471-213X-12-16 (PMC3426487; doi:10.1186/1471-213X-12-16)
Supplement: Additional file 1 — Figure S1.Protein alignment of human HDAC4 with zebrafish Hdac4. Functional domains are shaded as follows: CtBP-binding domain (pink), MITR-binding domain (yellow), serine residues associated with nuclear export by by chaperone 14-3-3 (blue), nuclear localization signal (grey), deacetylase domain (green), nuclear export signal (purple). Alignment was performed using SIM Alignment software. [file 1471-213X-12-16-S1.pdf]

78.1% identity in 1057 residues overlap; Score: 4090.0; Gap frequency: 3.7%

```
Human        32 VDVATALPLQVAPSAVPMDLRLDHQFSLPVAEPALREQQLQQELLALKQKQQIQRQILIA
zebrafish     2 VDVSVALPLQVPPSAIPMDLRVDHQFALAPTDPAQREHQLQQELLALKQKQQIQRQLLIE
                ***  ****** *** ***** **** *    ** ** ****************** **

Human        92 EFQRQHEQLSRQHEAQLHEHIKQQQEMLAMKHQQELLEHQRKLERHRQEQELEKQHREQK
zebrafish    62 EFQRQHEQLSRQHEAQLQEHVKHQQDLLALKHQQELLEHQRKLERHRQEQEMEKQQREQK
                ***************** ** * **  ** ********************* *** ****

Human       152 LQQLKNKEKGKESAVASTEVKMKLQEFVLNKKKALAHRNLNHCISSDPRYWYGKTQHSSL
zebrafish   122 LQLLKNKERGQESAVASTEVKMRLQEFVLNKKKALAQRSLNHCLPSDPRYWYGKTQHSSL
                ** ***** * *********** ************* * ****  ***************

Human       212 DQSSPPQSGVSTSYNHPVLGMYDAKDDFPLRKTASEPNLKLRSRLKQKVAERRSSPLLRR
zebrafish   182 DQSSPPQTGIST-YNHPVLGMYDPKDDFPLRKTASEPNLKLRSRLKQKVSERRSSPLLRR
                ******* * ** ********** ************************* **********

Human       272 KDGPVVTALKKRPLDVTDSACSSAPGSGPSSPNNSSGSVSAENGIAPAVPSIPAETSLAH
zebrafish   241 KDGPITTA-KKRSLDMAESACSSAPGSGPSSPNNSSNNITNENGIAGSISNSAVEPSLAH
                ****  ** *** **   ******************     *****        * ****

Human       332 RLVAREGSAAPLPLYTSPSLPNITLGLPATGPSAGTAGQQDAERLTLPALQQRLSL----
zebrafish   300 RLTGREGPVSQLSLYTSPSLPNITLGLPATGPSSVASGQQDGDRMAVPGLQPGIPITTPF
                **  ***    * ********************    ****  *   * **

Human       388 FPGTHLTPYLSTSPLERDGGAAHSPLLQHMVLLEQPPAQAPLVTGLGALPLHAQSLVGAD
zebrafish   360 MPGAHLPSYLAASSLERESGSAHNTLLQHMVLLEQSAAQNSLVAGLSGVPLQ--------
                 ** **  **  * *** *  **  **********  **  ** **   **

Human       448 RVSPSIHKLRQHRPLGRTQSAPLPQNAQALQHLVIQQQHQQFLEKHKQQFQQQQLQMNKI
zebrafish   412 --SHSIHKLRQHRPLGRTQSAPLPQNSQALQQLVVQQQHQQFLEKHKQQFQQQQLHISKM
                  * ********************** **** ** ********************   *

Human       508 IPKPSEPARQPESHPEETEEELREHQALLDEPYLDRLPGQKEAHAQAGVQVKQEPIESDE
zebrafish   470 MVKPSEPNRQHESHPEETEEELREHQGLGDP--ADPLPH--------GVTIKQEPPDEQD
                  ***** ** *************** * *    * **         **  ****

Human       568 EEAEPPREVEPGQRQPSEQELLFRQQALLLEQQRIHQLRNYQASMEAAGIPVSFGGHRPL
zebrafish   520 EDFQQ-------RERQAEEELLFRQQALLLEQQRIHQLRNYQASMEAAGLSVSFPVHRPL
                *                * ******************************  ***  ****

Human       628 SRAQSSPASATFPVSVQEPPTKPRFTTGLVYDTLMLKHQCTCGSSSSHPEHAGRIQSIWS
zebrafish   573 SRAQSSPASATFPIVVQEPPAKPRFTTGLVYDTLMQKHQCMCGNSNIHPEHAGRIQSIWS
                *************  ***** ************** **** ** *  *************

Human       688 RLQETGLRGKCECIRGRKATLEELQTVHSEAHTLLYGTNPLNRQKLDSKKLLGSLASVFV
zebrafish   633 RLQETGLRGQCECIRGRKATLEELQTVHSEAHVLLYGTNPL-RQKLDS-----SVTPMFV
                ********* ********************** ******** ******     *    **

Human       748 RLPCGGVGVDSDTIWNEVHSAGAARLAVGCVVELVFKVATGELKNGFAVVRPPGHHAEES
zebrafish   687 RLPCGGIGVDSDTIWNEVHSSSAARLAVGSVVDLVFKVASGELRNGFAVVRPPGHHAEES
                ****** *************  ******* ** ****** *** ****************

Human       808 TPMGFCYFNSVAVAAKLLQQRLSVSKILIVDWDVHHGNGTQQAFYSDPSVLYMSLHRYDD
zebrafish   747 TPMGFCYFNSVAIAAKLLQQRLNVSKILIVDWDVHHGNGTQQAFYSDPNVLYLSLHRYDD
                ************ ********* ************************* *** *******

Human       868 GNFFPGSGAPDEVGTGPGVGFNVNMAFTGGLDPPMGDAEYLAAFRTVVMPIASEFAPDVV
zebrafish   807 GNFFPGSGAPDEVGIGPGVGFNVNMAFTGGLEPPMGDADYLAAFRSVVMPIANEFSPDVV
                ************** **************** ****** ****** ****** ** ****

Human       928 LVSSGFDAVEGHPTPLGGYNLSARCFGYLTKQLMGLAGGRIVLALEGGHDLTAICDASEA
zebrafish   867 LVSSGFDAVEGHPPPLGGYKLTAKCFGYLTKQLMGLAGGRLVLALEGGHDLTAICDASEA
                ************* ***** * * **************** *******************

Human       988 CVSALLGNELDPLPEKVLQQRPNANAVRSMEKVMEIHSKYWRCLQRTTSTAGRSLIEAQT
zebrafish   927 CVSALLGNELDPIPEDILQQRPNANAIQSMEKVLEVQSKYWRSLQRSVSTLGYSLREAQR
                ************ **  *********  ***** *  ***** ***  ** * ** ***

Human      1048 CENEEAETVTAMASLSVGVKPAEKRPDEEPMEEEPPL
zebrafish   987 CENEEAETVTAMASLSVANKHMGKRPEEEPMEEEPPL
                *****************  *   *** **********
```
